# Cantharidin suppresses hepatocellular carcinoma development by regulating EZH2/H3K27me3-dependent cell cycle progression and antitumour immune response

**DOI:** 10.1186/s12906-023-03975-0

**Published:** 2023-05-18

**Authors:** Jia Yan, Xiu ling Deng, Shi qi Ma, Yu hui Li, Yu min Gao, Gui tao Shi, Hai sheng Wang

**Affiliations:** 1 School of Basic medical, Inner Mongolia Medical University, Hohhot, Inner Mongolia China; 2 School of Public health, Inner Mongolia Medical University, Hohhot, Inner Mongolia China; 3 Inner Mongolia Hospital of Traditional Chinese Medicine, Hohhot, Inner Mongolia China; 4 Affiliated Hospital of Inner Mongolia Medical University, Hohhot, Inner Mongolia China

**Keywords:** Cantharidin, Hepatocellular carcinoma, Network pharmacology, H3K27me3, Immune response

## Abstract

**Background:**

Cantharidin (CTD) is a major ingredient of cantharis (*Mylabris phalerata* Pallas) and has been used extensively in traditional Chinese medicines. It has been shown to exhibit anticancer activity in multiple types of cancer, especially hepatocellular carcinoma (HCC). However, there is no systematic study on the relationships among the regulatory networks of its targets in HCC therapy. We focused on histone epigenetic regulation and the influence of CTD on the immune response in HCC.

**Methods:**

We performed a comprehensive analysis of novel CTD targets in HCC based on network pharmacology and RNA-seq approaches. The mRNA levels of target genes were analyzed by qRT-PCR, and the corresponding protein levels were confirmed using enzyme-linked immunosorbent assay (ELISA) and immunohistochemical staining (IHC). ChIP-seq data were visualized by IGV software. The associations of gene transcript levels with the cancer immune score and infiltration level were investigated using TIMER. In vivo, the H22 mouse model of hepatocellular carcinoma was established by treatment with CTD and 5-Fu. The immune cell proportions in the blood were elevated in model mice, as shown by flow cytometry.

**Results:**

We identified 58 targets of CTD, which were involved in various pathways in cancer, including apoptosis, the cell cycle, EMT and immune pathways. Moreover, we found that 100 EMT-related genes were differentially expressed after CTD treatment in HCC cells. Interestingly, our results confirmed that the EZH2/H3K27me3 -related cell cycle pathway is a therapeutic target of CTD in antitumour. In addition, we evaluated the influence of CTD on the immune response. Our data showed that the significantly enriched gene sets were positively correlated with the chemokine biosynthetic and chemokine metabolic modules. The proportions of CD4+/CD8 + T cells and B cells were increased, but the proportion of Tregs was decreased after treatment with CTD in vivo. Moreover, we found that the expression of the inflammatory factor and immune checkpoint genes PD­1/PD-L1 was significantly reduced in the mouse model.

**Conclusion:**

We performed a novel integrated analysis of the potential role of CTD in HCC treatment. Our results provide innovative insight into the mechanism by which cantharidin exerts antitumour effects by regulating target genes expression to mediate apoptosis, EMT, cell cycle progression and the immune response in HCC. Based on the effect of CTD on the immune response, it can be used as a potential effective drug to activate antitumour immunity for the treatment of liver cancer.

**Supplementary Information:**

The online version contains supplementary material available at 10.1186/s12906-023-03975-0.

## Introduction

Hepatocellular carcinoma (HCC) is one of the most common malignant tumours with a high rate of recurrence and mortality worldwide [1, 2]. At present, the survival of patients with advanced HCC remains intractably low due to the latency period and insensitivity to chemotherapy [3]. In recent years, more and more studies have indicated that Chinese herbal medicines have obvious benefits in the treatment of solid tumours, including HCC [4, 5]. Therefore, exploring the molecular mechanisms of these medicines will provide more precise direction for the treatment of liver cancer.

Cantharidin is a sesquiterpenoid bioactive substance extracted from cantharis (*Mylabris phalerata* Pallas), which is a traditional Chinese medicinal preparation. The chemical formula of cantharidin is C_10_H_12_O_4_, and the chemical structure is shown in Supplementary Figure S1. Cantharidin and related pharmaceutical preparations have been reported to have anticancer activity [6–8]. For example, CTD is a major ingredient of Aidi injection (China Food and Drug Administration Z52020236) [7], which is approved for clinical use in China in 2002 [6, 7], and is widely used for the treatment of several cancer types, including liver cancer [7, 8], lung cancer [9], colorectal cancer [8], and gastric carcinoma [10].

Numerous studies have proposed mechanisms by which cantharidin exhibits antitumour activity by affecting the synthesis of RNA and DNA to induce DNA damage and apoptosis, and suppress cell proliferation in several types of tumours [11]. Several studies have indicated that CTD treatment suppresses cell proliferation via JAK2/STAT3, PI3K/Akt, and p38 MAPK pathway regulation [12–14]. Moreover, CTD can function as an inhibitor of protein phosphatase 2 A (PP2A) to induce DNA damage and apoptosis [15]. Recently, it has been reported that CTD could increase the chemosensitivity of liver cancer cells to upregulate the expression of KDM4A and lead to H3K36me3-dependent DNA damage in HCC [16]. However, new therapeutic targets and detailed pharmacological mechanisms remain to be further elucidated. In our study, integrated analysis of the pharmacological mechanism of cantharidin, its epigenetic regulation, especially H3K27me3-related histone modification, and its effects on the immune response pathway were performed to determine its utility for the treatment of HCC.

Histone methylation plays a crucial role in regulating chromatin formation and gene expression to affect pathological processes in various cancers [17–19]. Enhancer of Zeste Homolog 2 (EZH2) is a core component of Polycomb Repressive Complex 2 (PRC2), which has been reported to play important role in oncogenesis, tumour growth and metastasis in numerous malignant tumours by regulating gene expression through trimethylation of H3K27 [17]. EZH2 could be a potential therapeutic target in HCC. Moreover, it has been confirmed that EZH2 can regulate the expression of inflammatory cytokines and chemokines [18, 19].

We performed a comprehensive integrated analysis of the pharmacology and molecular mechanisms of cantharidin in HCC. In particular, we identified new potential targets of CTD and mainly focused on EZH2-H3K27me3-related gene expression networks in the context of HCC therapy. Moreover, we investigated the association of cantharidin with aspects of the immune response, especially chemokine-, inflammatory factor- and immune checkpoint gene-related pathways. Our findings provide new insight into the mechanism of cantharidin in antitumour therapy for HCC.

## Materials and methods

### Pharmacological regulatory network analysis

CTD-related targets were assessed based on data from drug target prediction platforms, including the Herb, SWISS-PROT, and Stitch databases. Then, the PPI network of CTD and its targets was constructed via Cytoscape 3.2.1 software. Moreover, we used the Venn diagram package to confirm the key targets, key module genes, drug targets, and disease targets of HCC based on the differentially expressed gene sets after CTD treatment in HCC cells and information about CTD targets obtained from databases. The hub gene set was obtained based on a bioinformatics approach [20, 21] Furthermore, the involvement of these genes in functions and pathways were confirmed based on Gene Ontology (GO) and Kyoto Encyclopedia of Genes and Genomes (KEGG) [22, 23].

### Reagents and cells

Cantharidin was purchased from Sigma-Aldrich, China (C7632, purity ≥ 98%). H22 cell was obtained from the Cell Bank of Chinese Academy of Sciences. 5-FU was purchased from Solarbio, China.

### RNA-seq analysis

Total RNA was obtained by the TRIzol method according to the manufacturer’s instructions. RNA-seq was performed by the HiSeqTM 2500 system (Illumina) at Novogene Corporation Inc. The difference in the expression matrix based on the sequencing data was analysed using the R package. Genes with a mean signal |logFC| ≥1, and *P* value ≤ 0.05 were considered to be significantly differentially expressed genes (DEGs) for further analysis. All the differentially expressed genes are listed in the Additional file 1. Functional and pathway enrichment analyse were performed based on GO terms, KEGG pathways and gene set enrichment analysis (GSEA). GSEA hallmark gene sets were obtained from MSigDB.

### ChIP-seq data analysis

The H3K27me3 ChIP-seq data of HepG2 cells were obtained from the Cistrome DB database. The Venn diagram package was used to identify the key genes based on a bioinformatics approach [24, 25]. The overlap of the H3K27me3 ChIP and RNA-seq data was confirmed. The levels of H3K27me3 enrichment in gene promoter regions were visualized by IGV2.13.2 software.

### Correlation analysis of immune checkpoints

Immune analysis of CXC and CCL chemokines in LIHC was performed using the ESTIMATE algorithm. The correlations between chemokine levels and immune cell infiltration, the immune score and the stromal score were assessed by Spearman correlation analysis. A *p*-value < 0.05 was considered significance threshold.

### Immune infiltration analysis

The correlations between chemokine levels and the infiltration of the 25 immune cell types were evaluated in LIHC based on the GSCA (Gene Set Cancer Analysis) database. The GSVA package was used to analyse the levels of immune cell infiltration in the groups with threshold criteria of a false discovery rate (FDR) < 0.05 and *p* value < 0.05. A *p*-value of < 0.05 was considered significance threshold.

### Establishment of the animal model and in vivo experiment

The H22 mouse model of hepatocellular carcinoma was constructed to confirm the antitumour function of cantharidin in vivo. The Male BALB/c mice (5–6 weeks old) weighing 20 ± 2 g was purchased from Beijing Viton Lihua Experimental Animal Technology Co., LTD. We randomly divided the H22 tumour-bearing mice into five groups (n = 10 per group): the control (oral administration of saline solution), groups treated with different doses of CTD (low (0.25 mg/kg), middle (0.5 mg/kg) and high (1 mg/kg)), and a group treated with 5-Fu (0.5 ml). CTD was obtained from Sigma-Aldrich, and its purity was ≥ 98.0%. The mice were weighed every 2 days. After two weeks, all the mice were fasted for 12 h, anesthetized by administration with 10% ULTANE per 10 g/ml intraperitoneal injection (Beijing Bailingwei Technology Co., LtD.) and sacrificed by cervical dislocation. The tumours were obtained and weighed before washing in normal saline. The inhibition rate (%) of tumour was calculated (mean tumor weight of model group - mean tumour weight of treatment group)/mean tumour weight of model group × 100%) and recorded.

### Haematoxylin and eosin (H&E) staining

The tumour tissues were fixed with 4% paraformaldehyde, dehydrated with 75% ethanol and embedded in paraffin. The samples were cut into 4 μm-thick sections and dewaxed with xylene. Then, they were rehydrated in an ethanol gradient and stained using haematoxylin. Next, the samples were immersed first in hydrochloric acid alcohol differentiation solution and then in eosin solution. Next, the sections were observed and photographed using a microscope.

### Analysis of immune cells in blood by flow cytometry

Blood samples (100 µL) were collected from mice in each group 20 days after drug treatment. Then, the red blood cells were lysed by ACK lysing buffer (Solarbio, China). Flow cytometry was used to confirm the percentages of CD4 + T (CD3 + CD4+), CD8 + T (CD3 + CD8), B (CD19+), and Treg (CD25+) cells in peripheral blood (Beckman, USA). Treg cells were detected from the CD4 + T cell population. The CD25 + phenotype represented Treg cells. Monocytes in peripheral blood were used as the total analysis population, and the CD19 + phenotype represented B cells. All the data were analysed using FlowJo7.6 software.

### Enzyme-linked immunosorbent assay (ELISA)

Tissue (5 mg) was collected, and PBS buffer was added. Then the tissue homogenate was prepared by a homogenizer. The concentrations of IL-2, IL-4, IL-10, TNF-α, and IFN-γ were determined by ELISA kits (Bio Legend, USA) according to the manufacturer’s instructions. The concentrations of IL-2, IL-4, IL-10, TNF-α, and IFN-γ were calculated using a microplate reader (Bio-Rad, Italy). A *P* value of < 0.05 was regarded as statistically significant, * *p* < 0.05.

### Immunohistochemical staining (IHC)

The expression of PD1 and PDL1 was confirmed using an IHC assay. Sections were obtained and incubated first with anti-PD1 and anti-PDL1 antibody for 16 h at 4 °C and then with a secondary antibody for 2 h at room temperature. Then, the secondary antibody was detected using a Tissue Staining Horseradish Peroxidase (HRP)-Diaminobenzidine (DAB) Kit. In addition, the protein expression levels of CCNA2, CENPA, BRCA2, RFC3, PSME3, and TERF2 in patients with HCC were obtained from the HPA database.

### Real-time RT-PCR

Total RNA was extracted using RNA Plus Reagent (Takara, China) and reverse transcribed into cDNA using a PrimeScript RT Reagent Kit (Takara, China). A SYBR Premix Ex Taq II (Takara, China) Kit was used to measure the mRNA level of each gene. The assay was performed in the CFX6 thermal cycler (Bio Rad, USA). Target genes expression were normalized to GAPDH expression to evaluate their expressions. All experiments were repeated more than three times. The results were considered to be statistically significant when the value of *p* was < 0.05, * *p* < 0.05, ** *p* < 0.01.

### Statistical analysis

Data are summarized as the mean ± standard deviation (SD) values. Student’s *t* test was used to analysed the differences between two groups, while one-way ANOVA followed by the Tukey’s post-hoc test was performed to evaluate the statistical significance among multiple groups. Differences were considered to be statistically significant when the value of *p* was < 0.05.

## Results

### Prediction and enrichment analysis of potential therapeutic targets and pathways of cantharidin in HCC

**Fig. 1:**
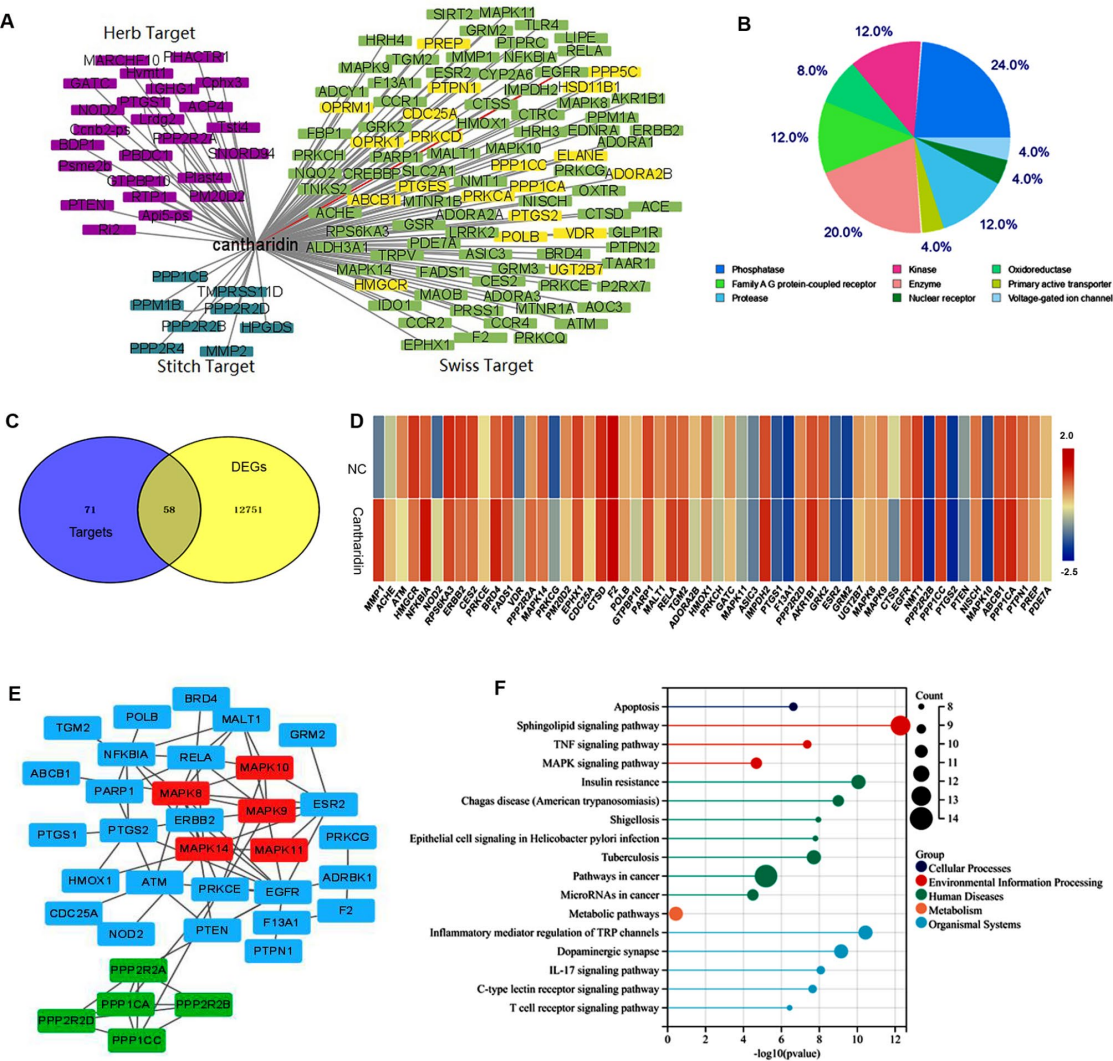
The targets network of cantharidin in HCC. **(A)** Network diagram of pharmacological regulation of cantharidin targets in HCC. Purple represents the targets of cantharidin from Herb database, dark green shows the targets of cantharidin from Sitch database; green and yellow are the targets from Swiss database. The probability yellow marker gene > 0.03, green marker gene < 0.03. **(B)** Statistical analysis protein properties of targets from different database. **(C)** The venn diagram shows the intersection of targets gene sets from three database and differentially expressed genes from cantharidin treated HepG2 cell. **(D)** Heat map shows the gene expression of predicted cantharidin targets in HepG2 cell. **(E)** PPI network of cantharidin targets in HCC. Red and green represent two hub networks in cantharidin targets. **(F)** Pathways with significant enrichment of target genes of cantharidin in HCC

To dertermine the pharmacological mechanism of cantharidin in HCC, the targets of cantharidin were obtained from the Herb, Stitch, and SWISS-PROT databases, and the network pharmacology map was constructed based on the interactions between these targets and LIHC (Fig. 1A). Moreover, protein function annotation indicated that these targets of CTD were mainly enzymes, phosphatases, kinases, and oxidoreductases (Fig. 1B). To further explore the therapeutic targets of CTD in HCC, the HepG2 cells were treated with cantharidin and subjected to RNA-seq analysis. Subsequently, integrated analysis was performed based on the RNA-seq results and database information. A Venn diagram was used to confirm the key targets of CTD based on the targets predicted by the databases and the differentially expressed genes (DEGs) identified by RNA-seq in HCC (Fig. 1C). In total, 58 DEGs overlapped with targets from the databases, suggesting that these genes are the key targets of CTD in HCC (Fig. 1D). Furthermore, the interaction network of these target proteins was constructed via the STRING database. As shown in Fig. 1E, MAPK8/9/10/11/14, the PP1 and PP2 phosphatases, NFKB1A, and EGFR were the hub targets of CTD (Fig. 1E). Additionally, the KEGG enrichment analysis results indicated that these genes were significantly enriched in pathways in cancer. Moreover, these genes were also involved in the TNF signalling pathway, the MAPK signalling pathway, inflammatory mediator regulation of TRP channels, the IL-17 signaling pathway, the T cell receptor signaling pathway, and the apoptosis pathway (Fig. 1F). Considering these results collectively we speculated that cantharidin exerts its therapeutic effect by regulating these target genes and their related signalling pathways to inhibit the development of liver cancer.

**Fig. 2:**
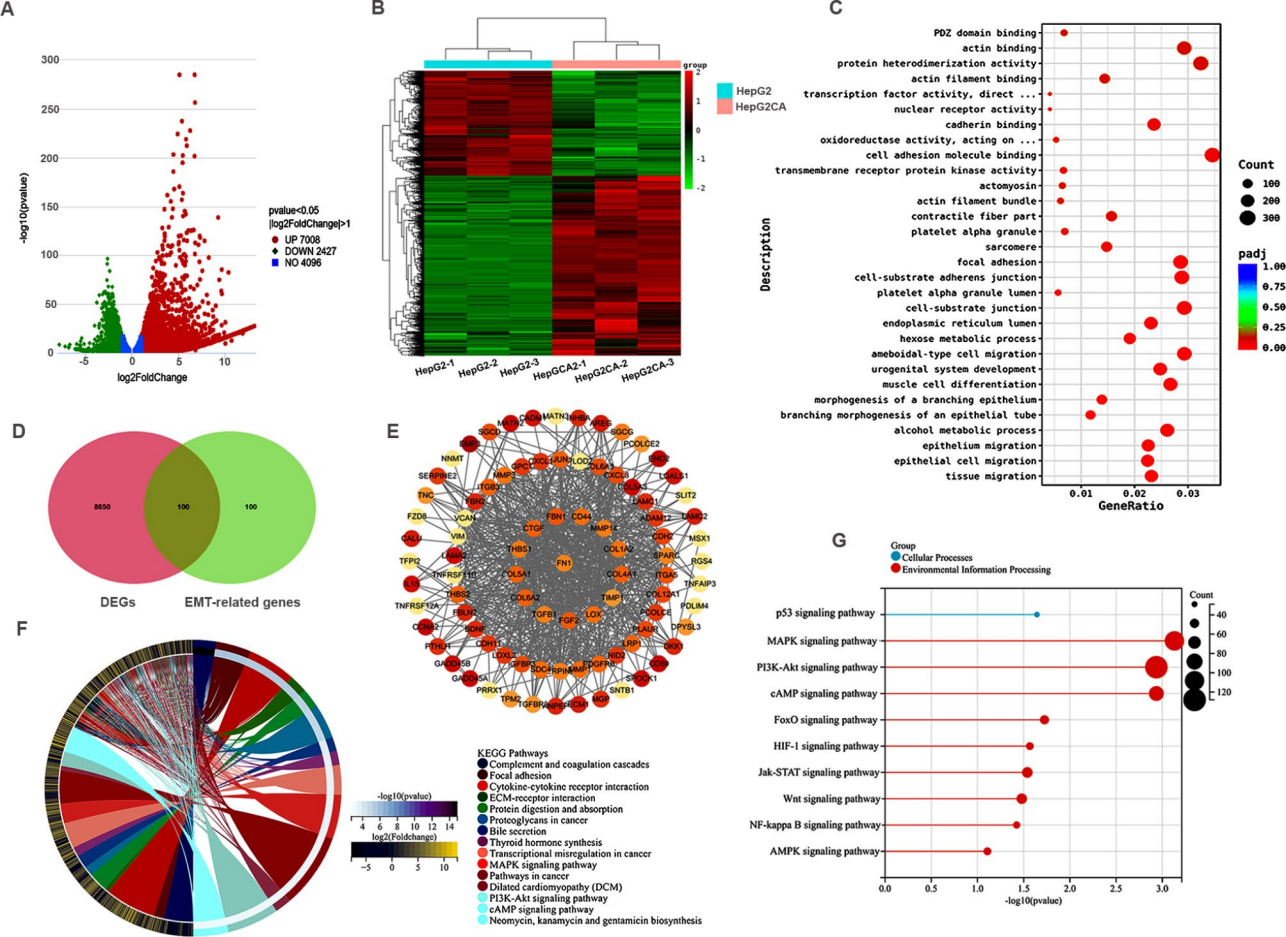
Differentially expressed genes and their function enrichment analysis in cantharidin treated HepG2 cell. (A, B) volcano plot and heat map visualization of the differentially expressed genes after cantharidin treatments in HepG2. Red represents upregulation genes; green represents downregulation genes. **(A)** Volcano plot of genes. **(B)** Heat map of differentially expressed genes that logFC > 1, <-1, *p* < 0.05. **(C)** GO enrichment of differentially expressed genes. **(D)** The EMT related genes after cantharidin treatment HepG2 cell was screened using venn diagram. **(E)** PPI network of EMT related genes. **(F)** KEGG enrichment of the differentially expressed genes after cantharidin treatment in HepG2 cell. **(G)** Top 10 significantly enriched signaling pathways in cantharidin treated HepG2 cell

To further explore the potential mechanism of cantharidin in HCC, we analysed our transcriptome data in depth. Based on threshold value of *p* < 0.05 and |log2FoldChange| > 1, a total of 7008 upregulated genes and 2427 downregulated genes were identified between HepG2 cells with and without cantharidin treatment (Fig. 2A). The expression of these genes was shown in Fig. 2B. Subsequently, GO and KEGG enrichment analyses were performed. These DEGs were significantly enriched in the terms of epithelium migration, epithelial cell migration, and tissue migration (Fig. 2C), suggesting that cantharidin likely inhibits cell metastasis by regulating EMT in liver cancer. Furthermore, the EMT gene set was obtained from the GSEA database and we found that 100 EMT-related genes were differently expressed after CTD treatment (Fig. 2D). Then, a PPI network of EMT-related genes was constructed according to the interaction relationships in the STRING database (Fig. 2E). The results indicated that cantharidin likely regulates these EMT-related genes to inhibit tumour cell growth and metastasis. Next, KEGG enrichment analysis was performed, and the results showed that the top 4 terms were “Cytokine-cytokine receptor interaction”, “ECM-receptor interaction”, “Transcription mis-regulation in cancer” and “Pathways in cancer” (Fig. 2F). Moreover, the top 10 cancer-related pathways were showed in Fig. 2G, including the MAPK, PI3K-Akt, cAMP, Jak-STAT, Wnt, NF-kappa B, AMPK and HIF-1 signalling pathways. Taken together, these finding indicate that CTD might inhibit HCC cell growth by regulating the above multiple pathways.

### EZH2/H3K27me3 is essential for cell cycle progression in cantharidin treated HCC cells

**Fig. 3:**
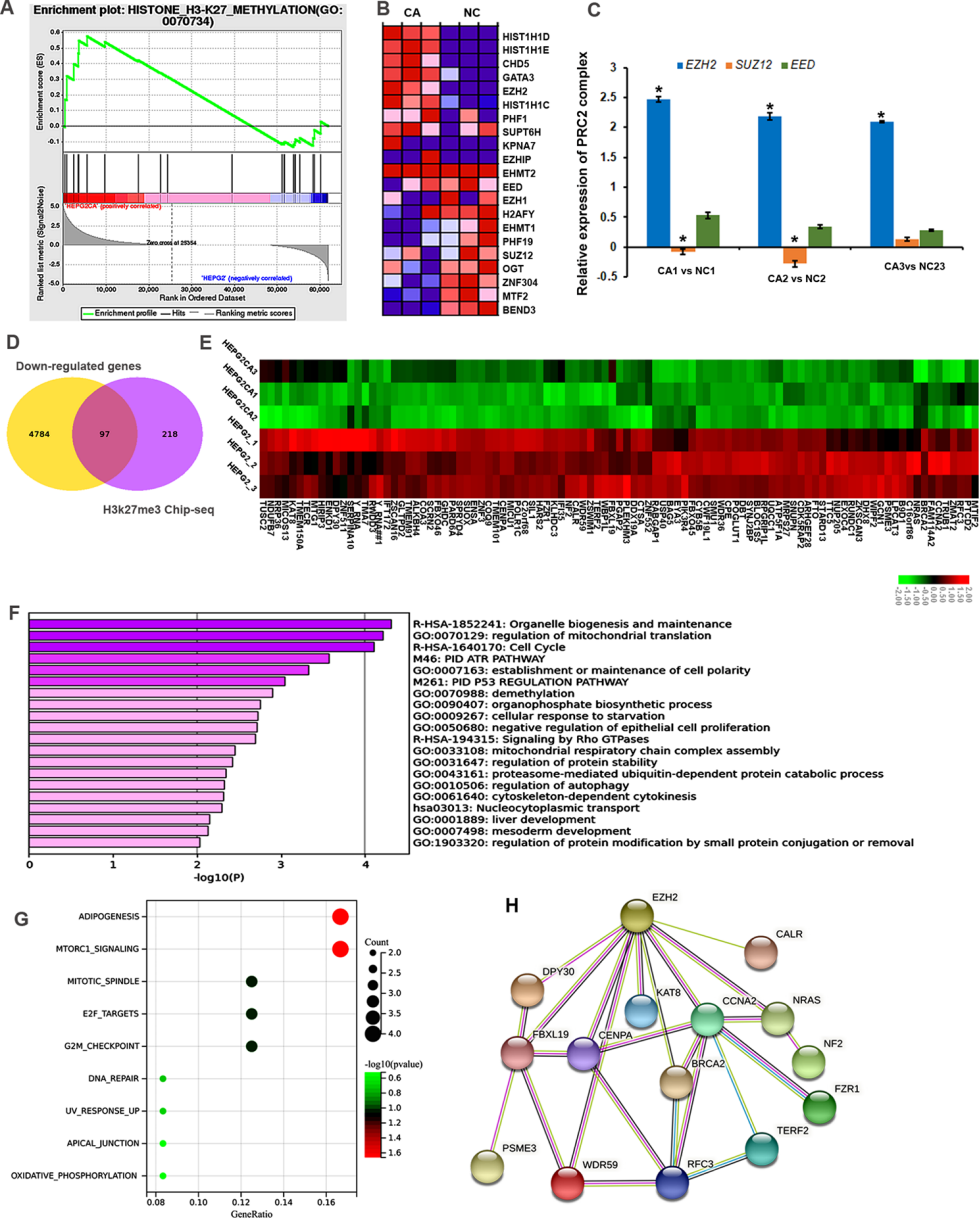
Cantharidin treatment is involved in EZH2/H3K27me3 related cell cycle pathway regulation. **(A)** GO functional annotation shows that cantharidin treatment is positively associated with H3K27me3 pathway based on GSEA analysis. **(B)** The heatmap shows H3K27me3 related genes expression in cantharidin treated HepG2 cell. Red represents up-regulation genes; blue represents down-regulation genes. **(C)** The expression of PRC2 complex genes, the key regulatory enzyme of H3K27me3, in cantharidin treated HepG2 cell. The *P < 0.05 was regarded as statistically significant. **(D)** Venn diagram shows the target genes of cantharidin downregulation genes and H3K27me3 significantly enriched genes from ChIP-seq results in HepG2 cell. **(E)** The heatmap shows the expression of 97 genes from venn analysis, which may be target genes of H3K27me3 in cantharidin treatment. Red represents up-regulation genes, green represents down-regulation genes. **(F)** Go enrichment analysis of the predicted target gene set. **(G)** Pathway analysis of the predicted target gene set. **(H)** PPI network of these target genes based on the STRING database results

Gene set enrichment analysis was performed to find promising therapeutic targets specific for CTD. Interestingly, the results showed that the DEGs in the CTD treated group were positively associated with H3K27me3 (Fig. 3A). EZH2 was significantly upregulated in CTD-treated samples compared to non-CTD-treated samples, implying that EZH2 may be the reason for the increase of H3K27me3 level after CTD treatment (Fig. 3B). Moreover, the RT-PCR results confirmed that EZH2 was upregulated in CTD treated HepG2 cells (Fig. 3C). To confirm the CTD-related H3K27me3-modified and repressed genes, we integrated our RNA-seq data from HepG2 cells and ChIP-seq datasets from the Cistrome Data Browser database. In total, 97 genes were occupied and downregulated by H3K27me3 in HepG2 cells (Fig. 3D, E). These data revealed that EZH2/H3K27me3 involved downstream target gene expression might play a critical role in the antitumour therapeutic effects of CTD.

We next uploaded the target genes to Metascape and found that these genes were significantly enriched in the regulation of organelle biogenesis and maintenance, mitochondrial translation, and cell cycle (Fig. 3F). Moreover, these genes were associated with cell cycle related pathways, such as mtorc1 signalling, mitotic spindle, E2F targets, and G2M checkpoint (Fig. 3G). To further confirm the relationships between these genes and EZH2, we constructed a PPI regulation network. The PPI results indicated that 7 proteins, DY310, FBXL19, CENPA, KAT8, CCNA2, NRAS, and CALR, directly interact with EZH2 (Fig. 3H). This information supported the notion that EZH2/H3K27me3 might directly suppress these genes to regulate the cell cycle during CTD therapy for liver cancer.

### Prediction of H3K27me3-related potential therapeutic targets of cantharidin in HCC

**Fig. 4:**
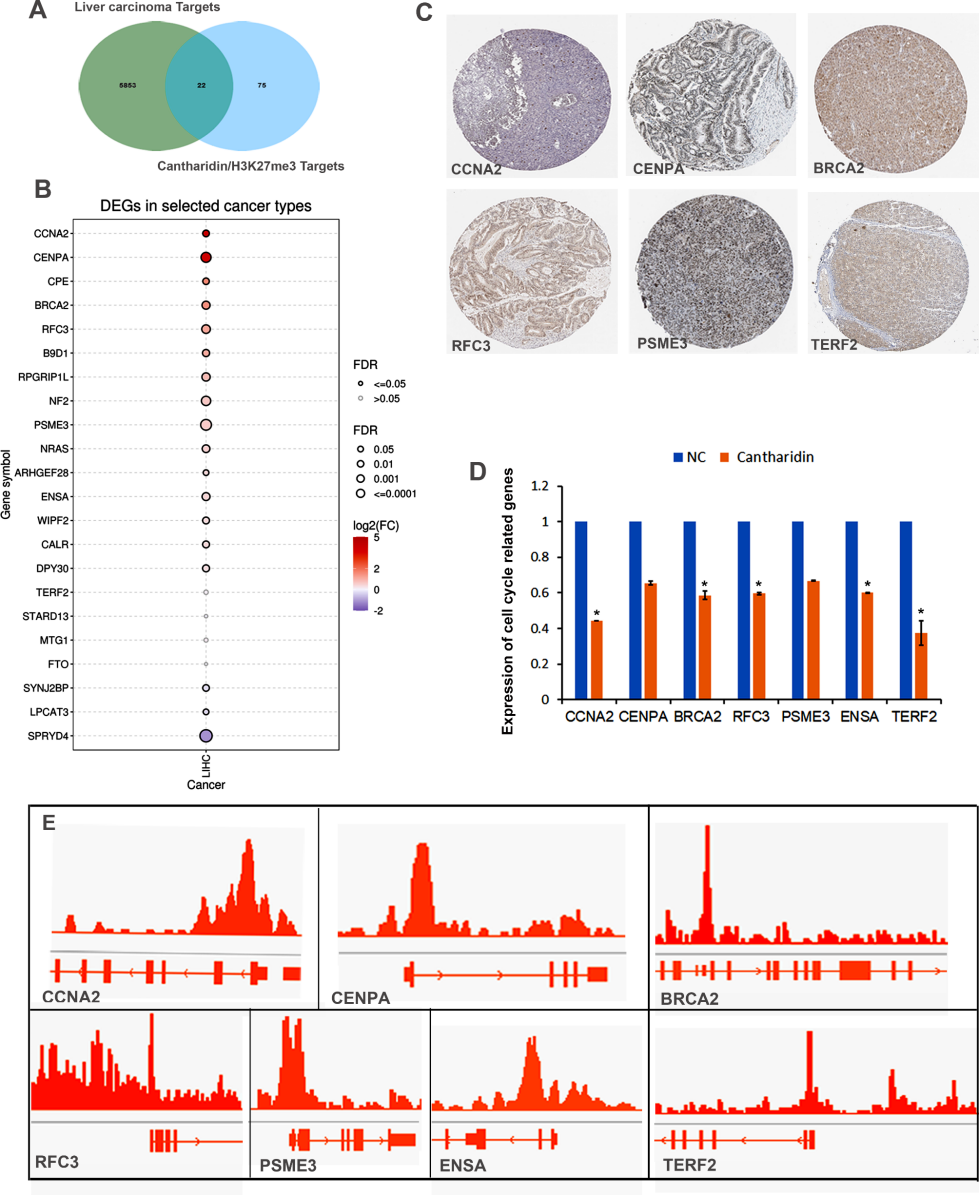
H3K27me3 related cantharidin treatment target prediction analysis in HCC. **(A)** Venn diagram shows the genes of liver carcinoma and H3K27me3 related cantharidin treatment targets. **(B)** The expression of these targets in LIHC. Red represents high expression; blue represents low expression. *P <* 0.05 is considered significant. **(C)** Proteins expression of significantly up-regulated genes in LIHC. **(D)** These genes expression in cantharidin treated HepG2 cell. The * *P* < 0.05 was regarded as statistically significant. **(E)** H3K27me3 is dramatically enriched in these genes promoters based on the ChIP-seq results in HepG2

We further searched disease targets of HCC and retrieved 5875 targets in the disease target database. The Venn diagram showed 22 co-target genes between the cantharidin/H3K27me3-related targets in HepG2 cells and the targets of liver cancer (Fig. 4A). Among them, *CCNA2*, *CENPA*, *CPE*, *BRCA2*, *RFC3*, *B9D1*, *RPGR1P1L*, *NF2*, and *PSME3* exhibited significantly elevated expression (fold change > 1.5) in the tissues of patients with HCC compared with the normal tissues (Fig. 4B). Moreover, their protein levels were also increased in the tissues of patients with HCC (Fig. 4C). Among of them, *CCNA2*, *CENPA*, *BRCA2*, *RFC3*, *PSME3*, *ENSA* and *TERF2* were significantly decreased (log2FC<-1) in CTD treated HCC cells (Fig. 4D). Moreover, the ChIP-seq results showed that H3K27me3 could be enriched in the promoter regions of these dramatically downregulated genes (Fig. 4E). Thus, we hypothesized that the EZH2/H3K27me3 cascade acts as a therapeutic target of CTD-related liver cancer treatment.

### Cantharidin regulated chemokine-related gene expression in liver cancer cells

**Fig. 5:**
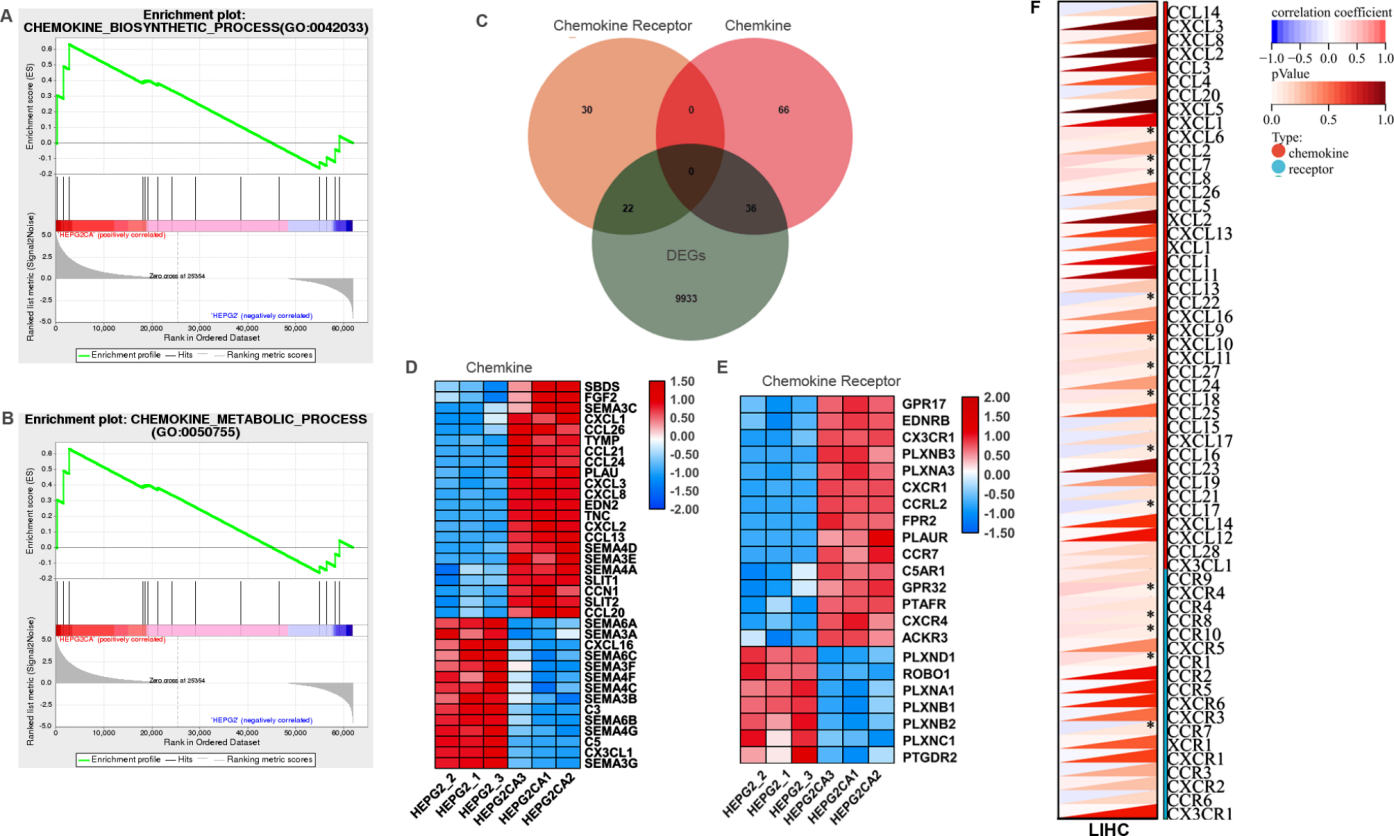
The cantharidin treatment affects chemokine related immune response. **(A, B)** GO functional annotation shows that cantharidin treatment is positively associated with chemokine biosynthetic and metabolic processes based on GSEA analysis. **(C)** Venn diagram shows that the differently expressed genes of chemokine and chemokine receptor in the cantharidin treated HepG2 cell. **(D, E)** The heatmap shows the expression of chemokine (D), and chemokine receptor **(E)** in the differently expressed genes of cantharidin treated HepG2 cell. **(F)** The heatmap shows the association of chemokine and chemokine receptor with EZH2 in LIHC, * *P* < 0.05 was regarded as statistically significant

Further GSEA analysis was performed on the overall expression data of CTD-related genes. The significantly enriched gene sets that positively correlated with CTD were the chemokine biosynthetic and chemokine metabolic modules (Fig. 5A, B). Then, we obtained chemokine- and chemokine receptor-related genes from the GSEA database. The Venn diagram shows that 36 chemokines and 22 chemokine receptors were differentially expressed in cantharidin-treated HepG2 cells (Fig. 5C). Notably, we found that *CXCL1/2/3/8* and *CCL20/21/24/26* were significantly upregulated, but *SEMA3/4/6* were downregulated after CTD treatment (Fig. 5D). Additionally, the levels of *CX3CR1*, *CXCR1*, *CCRL2, CCR7*, *CXCR4* and other chemokine receptors with atypical structures were increased and those of five members of the *PLXN* chemokine receptor family were dramatically decreased in the CTD treatment group (Fig. 5E). Furthermore, the correlations of EZH2 and chemokine were evaluated in HCC. As shown in Fig. 5F, EZH2 expression was significantly associated with that of several chemokines and chemokine receptors, including *CXCL6*, *CCL7*, *CCL8*, *CCL22*, *CXCL10*, *CCL27*, *CCL18*, *CCL16*, *CXCR4*, *CCR8*, *CCR10*, *CCR1*, and *CCR7*, in HCC.

**Fig. 6:**
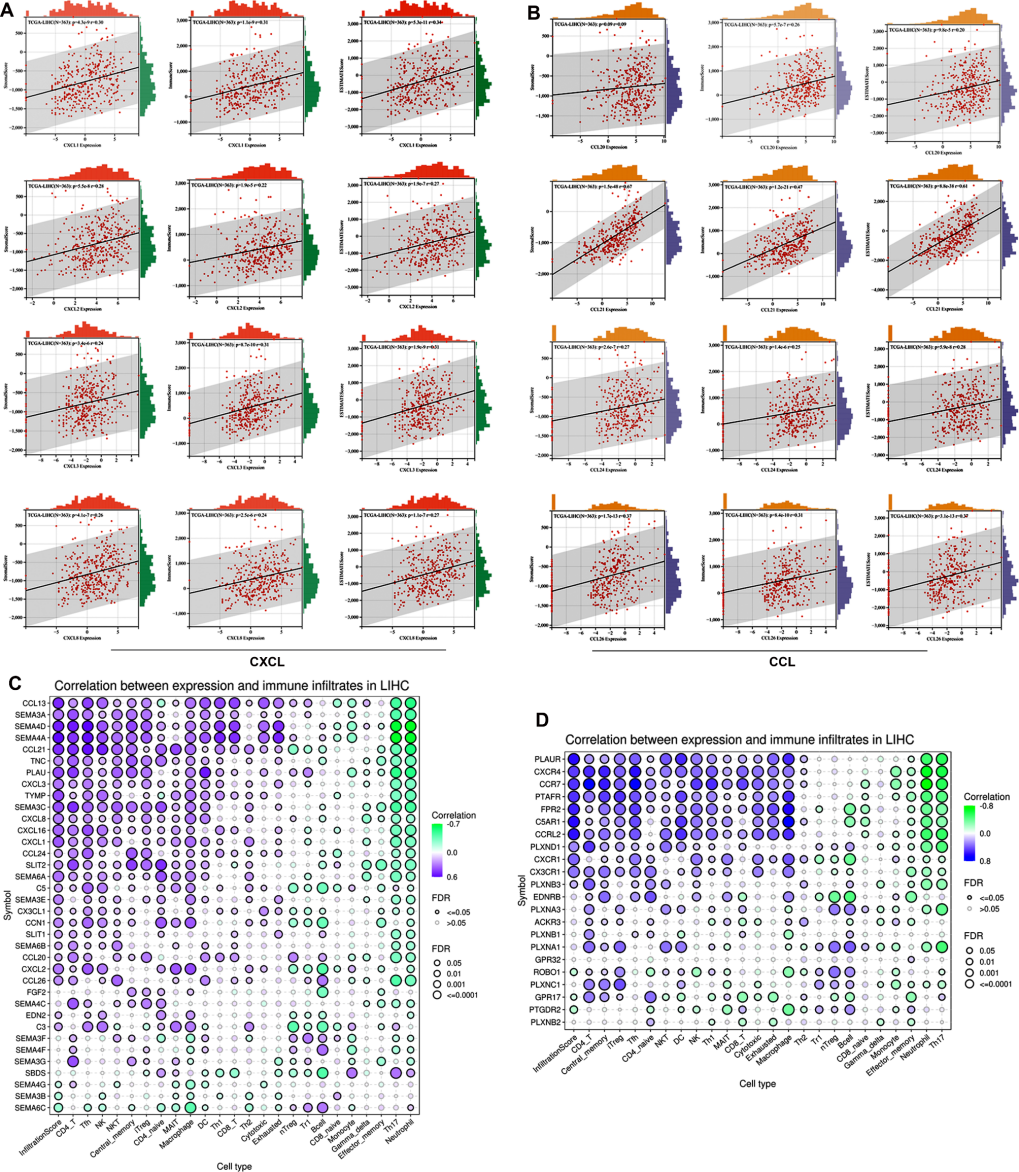
Cantharidin related chemokines were involved in tumor immune. (**A**, **B**) The correlations between CXC **(A)** and CCL **(B)** chemokines and immune score, stromal score, and ESTIMATE score. (**C**, **D**) The correlations between the expression of cantharidin related chemokines **(C)**, chemokine receptors **(D)** and the immune infiltration levels of 25 immune cells, which were analyzed by using the GSCA tools. Purple represents a positive correlation; green represents a positive correlation. * *P* < 0.05 and # FDR < 0.05 were regarded as statistically significant

In addition, the expression of CXC and CCL chemokines involved in the immune response was elevated. We found that the levels of the CTD-related chemokines *CXCL1/2/3/8* and *CCL20/21/24/26* were significantly correlated with the stromal score and immune score (Fig. 6A, B). Moreover, the relationships between differentially expressed chemokines and immune cell infiltration were evaluated in LIHC. Most chemokines and chemokine receptor were positively associated with immune cells infiltration, including CD4 + cells, NK cells, macrophages, and Treg cells (Fig. 6C, D). Among these genes, the chemokines* SEMA4A*, *SEMA4D*, *CCL13*, and *CCL21* and the chemokine receptors* C5AR1*, *PLAUR*, *CCRL2*, *FPR2*, *PTAFR*, *CCR7 *and *CXCR4* may be critical genes for immune cell infiltration (Cor > 0.48). Integrated analysis with the transcriptome data indicated that CTD-related DEGs were associated with several immune response signalling pathways, such as MAPK, PI3K-AKT, NF-kappa B, and HIF-1α pathways. Therefore, CTD likely inhibits the progression of HCC by affecting chemokines involved immune cell trafficking and immune signalling responses.

### Cantharidin inhibits tumour growth and enhances antitumour immunity in vivo

**Fig. 7:**
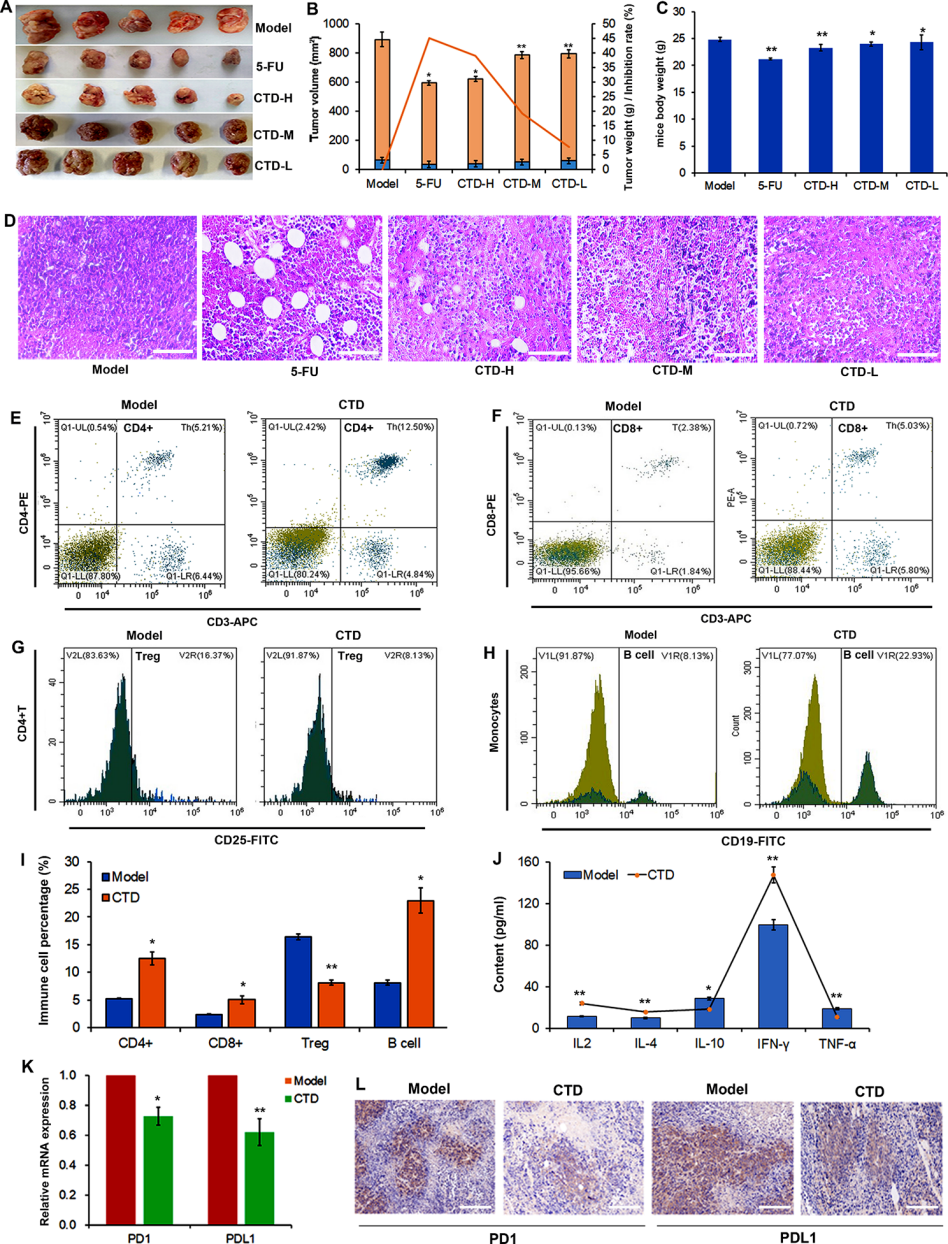
The cantharidin improves antitumor immunity in mice. **(A)** Cantharidin represses the tumor cell growth in *vivo.***(B)** Statistics of the tumor volume, weight, and inhibition rate in mice after therapy with cantharidin and 5-FU. Cantharidin and 5-FU have similar inhibitory effects on tumor growth. Data are presented as the mean ± SD (n = 3), * *P* < 0.05, ** *P* < 0.01 vs. the control group. **(C)** The mice body weight of different model groups. **(D)** The H&E staining of tumor tissues, including model, 5-FU, CTD high, middle, and low groups. (E-I) Proportion of CD4+ **(E)**, CD8+ **(F)**, Treg **(G)**, and B cell **(H)** in mice after therapy with cantharidin. To detect the immune cells, blood samples are collected from each treatment group. Cells are stained with anti-CD4, anti‐CD8, anti-CD25, and anti-CD19 antibodies and analyzed by flow cytometry. (I) Statistics of the frequency CD4+, CD8+, Treg, and B cell in mice blood. **(J)** Elisa assay confirms the expression of immune related genes, IL-2, IL-4, IL-10, TNF-γ, and TNF-α. **(K)** RT-PCR analyses the expression of *PD1* and *PD-L1* in the tumor tissues. The * *P* < 0.05, ** *P* < 0.01. *P* value of < 0.05 was regarded as statistically significant. **(L)** Immunohistochemistry and quantitative analyses the expression of PD1 and PD-L1 in the tumor tissues after therapy with cantharidin

To further confirm the antitumour function of CTD in HCC, we investigated the effect of CTD on tumour growth in vivo. The results showed that tumour volume and weight were significantly decreased in mice treated with a high concentration of CTD and 5-FU (Fig. 7A, B). The tumour inhibition rate of CTD was similar to that of 5-FU (Fig. 7B). However, the CTD group did not exhibit significantly altered total body weight compared with the 5-FU group (Fig. 7C). Moreover, the HE staining results showed that the number of cells was dramatically reduced and that the cell were loosely arranged. The numbers of necrotic and apoptotic cells were increased in the CTD group. As observed the 5-FU group, the number of abnormal vacuoles was increased in the high-concentration CTD group (Fig. 7D). Therefore, CTD could reduce tumour cell growth and promote apoptosis in vivo.

In addition, the CTD-mediated immune response was assessed. Flow cytometry was used to determine the proportions of CD4 + cells, CD8 + cells, Tregs and B cells in the blood of mice (Fig. 7E-H). The results showed that the proportions of CD4+/CD8 + T and B cells were increased after treatment with CTD, while the frequency of Tregs was decreased in the CTD group (Fig. 7I). These results indicated that CTD could inhibit tumour growth by affecting the immune cell distribution to enhance the antitumour immune response in HCC. We then proceeded to further investigate the molecular mechanism of CTD in the immune response. We measured the levels of inflammatory cytokines (TNF-α, IFN-γ, IL-2, IL-4, and IL-10) in the peripheral blood of model mice. As shown in Fig. 7J, IL-2, IL-4, and IFN-γ were elevated, but TNF-α and IL-10 were decreased following treatment with CTD. Next, the expression levels of the immune checkpoint genes PD­1/PD-L1 was were confirmed to investigate the effect of CTD on the immune response. The mRNA and protein expression levels of PD­1/PD-L1 were significantly reduced after CTD treatment (Fig. 7K, L). Therefore, it can be preliminarily concluded that CTD plays a critical role in the regulation of the immune response in exerting its anti-tumorigenic effects.

## Discussion

Cantharidin is the major component of the anticancer medicine obtained from cantharis and exhibits antitumour activity in several cancers, especially HCC [11]. Cantharidin and its derivatives are used in clinical antitumour therapy [26, 27]. However, the application of cantharidin is very limited due to its considerable hepatotoxicity. Recently, an increasing number of cantharidin derivative-related drugs have been developed, such as norcantharidin, magnesium demethylcantharidate, and methyl-cantharidimide [28–30], which exhibit lower toxicity and retain their anticancer activity. Norcantharidin has been reported to suppress c-Met-mTOR signalling to induce cell death in HCC [31]. The combination of magnesium demethylcantharidate and sorafenib results in *FOXO1* activation to inhibit cell invasion and metastasis [29]. Methyl-cantharidimide plays a critical role in reducing cisplatin resistance at *ABCB1*- and *ABCG2*-overexpressing cancer cells [32]. Therefore, a comprehensive analysis of the pharmacodynamic targets of cantharidin and its molecular mechanism is necessary for the application of cantharidin antitumour therapy in HCC.

In this study, we comprehensively analysed the potential pharmacodynamic targets of cantharidin in HCC. In total, 58 target genes were obtained and found to be significantly associated with pathways in cancer; among these genes, MAPKs and PP1/2 phosphatases were defined as the hub genes of cantharidin in HCC. This result is consistent with the previously reported targets of cantharidin. In addition, 100 CTD- associated and EMT-related genes were obtained, suggesting that CTD may inhibit invasion and metastasis by regulating the expression of these genes in HCC. Additionally, we innovatively analysed the effect of CTD on histone modification. Interestingly, we found that the DEGs related to cantharidin were positively associated with H3k27me3 modification. Moreover, this H3K27me3-enriched gene set was involved in cell cycle pathways. We proposed that CTD likely inhibits tumour progression by regulating the cell cycle pathway through the EZH2-H3K27me3-dependent gene expression network.

Cantharidin was also confirmed to play an important role in immune enhancement. CTD is involved in miR-214 modulated macrophage polarization to exert antitumour effects on HCC [33]. Moreover, norcantharidin has functions of the immune enhancement, suppression of platelet aggregation and inhibition of renal interstitial fibrosis [34, 35]. Our results indicated that chemokine biosynthetic and metabolic pathways are positively associated with cantharidin treatment. Most of the differentially expressed chemokines and chemokine receptors were significantly positively correlated with the infiltration of immune cells, especially CD4 + T cells, Tfh cells, NK cells, NKT cells, Tregs and macrophages. Consistent with the analysis results, the results of animal experiments indicated that the proportions of CD4+/CD8 + T and B cells were increased, but the proportion of Tregs was decreased after treatment with CTD.

The EZH2-related pathway has been reported to be involved in the regulation of various immune cells including macrophages [36]. EZH2 inhibitors suppress EZH2-mediated H3K27me3 levels and regulate macrophages by skewing the polarization of M2 towards effector M1 macrophages in CRC. EZH2 inhibits NK cell-mediated antitumour immunity by suppressing CXCL10 expression in an HDAC10-dependent manner [37]. It can decrease antigen-specific CD8 + T-cell proliferation, and IFNγ production to enhance antigen presentation and thus antitumour immunity in head and neck cancer [38]. Moreover, we found that EZH2 expression was significantly associated with that of several chemokines and chemokine receptors in HCC. Taken together, these findings indicate that CTD plays a critical role in antitumour immunity by regulating chemokine or EZH2 related pathways.

Recently, the therapeutic application of monoclonal antibodies targeting inhibitory pathways such as PD-1 and PD-L1 has been confirmed to generate meaningful improvement in the clinical outcome of HCC [39]. The combination of PD-L1 antibody (atezolizumab) with Avastin (bevacizumab) against vascular endothelial growth factor (VEGF) has been approved for clinical treatment of patients with advanced HCC [40, 41]. In this study, we firstly found that the PD­1/PD-L1 is dramatically repressed by CTD. Combined with the results that CTD was involve in chemokine, inflammatory factor expression, and immune cell proportion regulation. We speculated that CTD maybe regulated immune response pathway to inhibit immune cells infiltration and promote anti-tumor immune activation in HCC.

## Conclusion

**Fig. 8:**
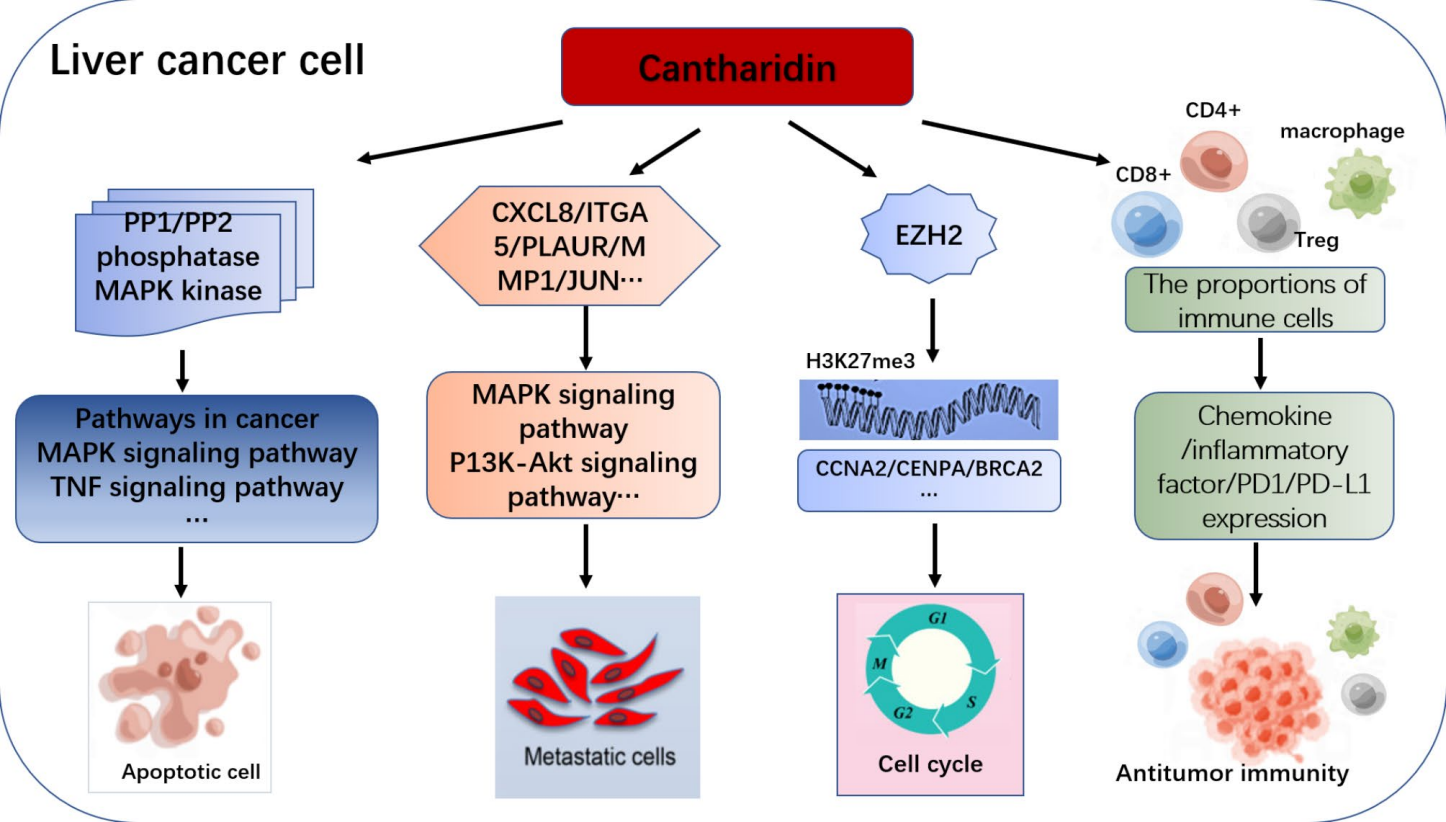
The function of cantharidin in HCC. Cantharidin is involved in regulation the cell apoptosis, EMT progresses, EZH2/H3K27me3 related cell cycle pathway and immune response to exert antitumor function in HCC.

In this study, we have not only identified the reported cantharidin targets MAPK and PPP1/2 phosphatase, but also confirmed several new targets of CTD in liver cancer. We revealed that MAPK, PPP1/2 phosphatase and EMT-related genes act as the hub genes of CTD network to regulate apoptosis and EMT processes. Moreover, CTD may represent a potent anticancer agent in HCC cells to regulated cell cycle via EZH2-H3K27me3 dependent genes expression network. In addition, we also confirm that CTD is involved in immune response, including the regulation of the chemokine related genes expression, inflammatory cytokines and immune checkpoint genes to repress tumor processes (Fig. 8). Our results provide a systematic view of the potential anticancer mechanism of cantharidin in HCC. Moreover, it indicates the potential research direction for innovative immunotherapy strategies in HCC. This will provide theoretical reference for the promotion of traditional Chinese medicine in clinical treatment for liver cancer.

## Electronic supplementary material

Below is the link to the electronic supplementary material.

**Additional File 1**: All differently expressed genes in HepG2 cell after cantharidin treatment

**Additional File 2**: **Figure S1** The chemical structure of cantharidin

## Data Availability

The datasets used in this article are publicly available as described in Materials and Methods. All result data was provided in the manuscript. All the gene expression raw data was provided in supplementary material. If anyone needs, they also can obtain raw data by contacting with the corresponding author.
