# Supplementary figures and images for: Cantharidin suppresses hepatocellular carcinoma development by regulating EZH2/H3K27me3-dependent cell cycle progression and antitumour immune response
Source: BMC Complement Med Ther. 2023 May 18;23:160. doi: 10.1186/s12906-023-03975-0 (PMC10193799; doi:10.1186/s12906-023-03975-0)

Figure S1. The chemical structure of cantharidin.

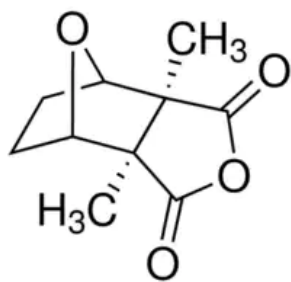

Supplement: Supplementary file 2 — Additional File 2: Figure S1 The chemical structure of cantharidin [file 12906_2023_3975_MOESM2_ESM.pdf]
